# Glaucoma Medication and Quality of Life after Phacoemulsification Combined with a Xen Gel Stent

**DOI:** 10.3390/jcm11123450

**Published:** 2022-06-15

**Authors:** Christian Pahljina, Stephanie Sarny, Lukas Hoeflechner, Thomas Falb, Gernot Schliessleder, Marlene Lindner, Domagoj Ivastinovic, Kaweh Mansouri, Ewald Lindner

**Affiliations:** 1 Department of Ophthalmology, Medical University Graz, 8036 Graz, Austria; christian.pahljina@hotmail.com (C.P.); stephanie@sarny.at (S.S.); lukas.hoeflechner@medunigraz.at (L.H.); thomas.falb@medunigraz.at (T.F.); gernot.schliessleder@medunigraz.at (G.S.); domagoj.ivastinovic@medunigraz.at (D.I.); 2 Department of Dentistry, Medical University Graz, 8036 Graz, Austria; marlene.lindner@medunigraz.at; 3 Glaucoma Research Center, Montchoisi Clinic, Swiss Visio, 1006 Lausanne, Switzerland; kwmansouri@gmail.com

**Keywords:** glaucoma, quality of life, surveys and questionnaires, minimal invasive glaucoma surgery, Xen gel stent

## Abstract

Glaucoma has a significant impact on quality of life. Here, we aimed to evaluate the influence of a reduction in glaucoma medications on quality of life and patient satisfaction after phacoemulsification combined with the Xen gel stent. We carried out a cross-sectional survey of patients who underwent phacoemulsification with the Xen gel stent at the Medical University of Graz, Austria. Quality of life was assessed using the German version of the Glaucoma Symptoms Scale (GSS)—questionnaire. Patients were also asked whether the operation reduced glaucoma medications and to indicate their overall satisfaction from 1 (totally discontented) up to 10 (totally contented). Questionnaires of 80 patients were evaluated. A total of 36 patients (45.0%) reported a reduction in glaucoma medications. Three items of the GSS were significantly better in patients who needed fewer glaucoma medications after the operation (“hard to see in daylight”, 75.0 ± 31.1 vs. 57.7 ± 39.1, *p* = 0.035; “hard to see in dark places”, 81.1 ± 28.7 vs. 54.9 ± 41.2, *p* = 0.002; and “halos around lights”, 88.3 ± 25.9 vs. 68.8 ± 38.6, *p* = 0.002). Patient satisfaction was significantly higher when the procedure led to a reduction in glaucoma medication (8.3 ± 2.0 vs. 6.8 ± 3.1; *p* = 0.034). The reported quality of life and patient satisfaction were significantly better when phacoemulsification with the Xen gel stent reduced the number of glaucoma medications needed.

## 1. Introduction

Glaucoma is a chronic neurodegenerative disease that negatively affects patients’ quality of life. The mere knowledge of having the disease has a negative effect on one’s quality of life, as has been shown in glaucoma suspects [1] and newly diagnosed glaucoma patients [2]. While the psychological burden of having the disease and the need for bothersome topical medications are more relevant in glaucoma’s early and moderate stages, visual impairment becomes more important in the more advanced stages [3]. In the early course of the disease, patients usually do not recognize visual field defects. A mean defect of as low as −18 dB is an important threshold for vision-related quality of life [4].

The quality of life of glaucoma patients is not only reduced by the disease itself but also by the treatment. Pain-related quality of life is affected in glaucoma patients [5] by concomitant ocular surface disease aggravated by topical anti-hypertensive medications [6] or glaucoma surgery [7]. While the prevalence of the ocular surface disease is between 5% and 30% in the general population, it increases to about 50% in glaucoma patients [8]. The long-term use of eye drops causes a decrease in conjunctival goblet cell density, squamous metaplasia, and Meibomian gland dysfunction [9]. Dry eye diseases causes visual disturbances that significantly impact one’s quality of life [10]. Tear film instability and ocular surface damage lead to fluctuating vision with blinking, glares, and blurred vision [11]. In glaucoma patients, dry eye disease is more prevalent when three or more glaucoma drugs are used [12]. The role of dry eye disease in glaucoma is widely recognized and should be addressed when evaluating medical or surgical treatment in order to maximize patient compliance [13].

Glaucoma surgery can drastically reduce the number of eye drops required. Recently, various techniques of minimally invasive glaucoma surgery have been introduced. The Xen^®^ gelatin ab interno implant (Abbvie, Allergan, IL, USA) connects the anterior chamber to the subconjunctival space and can be implanted standalone or in combination with cataract surgery [14]. The implantation of Xen^®^ can efficiently reduce intraocular pressure and lowers glaucoma medication count [15]. This study aimed to evaluate the quality of life in patients who have undergone minimal invasive glaucoma surgery with Xen^®^ in combination with cataract surgery.

## 2. Materials and Methods

We contacted patients who had undergone minimal invasive glaucoma surgery with Xen^®^ (Xen45, Allergan, IL, USA) in combination with cataract surgery at the department of ophthalmology of the Medical University of Graz between March 2014 and May 2018. This cross-sectional study was conducted according to the Declaration of Helsinki, and the local institutional review board approved the study protocol.

Prior to surgery, Goldmann Applanation Tonometry (GAT), best-corrected visual acuity (BCVA), slit-lamp biomicroscopy, standard automated perimetry (Interzeag Octopus 500, program G2), and optical coherence tomography (OCT, Heidelberg Spectralis^®^) were completed. Surgeries were performed in retrobulbar anesthesia. All Xen^®^ implants were performed with mitomycin C (MMC) (0.1 mg/mL) and inserted by an ab interno approach in the upper nasal quadrant. The MMC was applied subconjunctivally during a primary needling procedure. The postoperative management included unpreserved topical steroids and antibiotics for 5 weeks. One year after surgery, GAT and BCVA were repeated. For this study, data were acquired from clinical records.

The Glaucoma Symptom Scale (GSS) [16] is a questionnaire that includes 10 items grouped into two domains: non-visual symptoms (burning/stinging, tearing, dryness, itching, soreness/tiredness, feeling of something in the eye) and visual symptoms (blurry/dim vision, hard to see in daylight, hard to see in darkness, and halos around the lights). The GSS has been translated into many languages [17,18,19]. Each item can be scored from 0 to 100, where 100 means no symptoms. The total score is the mean of all scores. Non-visual symptoms and visual symptoms can be summarized. German questionnaires with a cover letter were sent to patients. We asked patients to only evaluate the operated eye. In addition to GSS, we also asked patients to score their general satisfaction with the surgery from 1 to 10 (where 10 means complete satisfaction), and we asked them to indicate whether the operation led to a reduction in glaucoma medications. Only questionnaires that had all items filled out were used for further analysis.

Before the questionnaire was sent out it had to be validated. The English version of the questionnaire was translated into German by two translators coming from different dialect groups in southern Austria. Discrepancies were reconciled. The German version was then back-translated into English by a non-ophthalmologist. There was a high equivalence with the original English version. The German version was then discussed by an expert panel and was handed out to consecutive glaucoma patients and healthy controls. Patients had to complete the questionnaire at two different appointments. The test-retest reliability for the German version was high and the questionnaire had a good discriminatory power between patients and healthy controls (*p* < 0.05).

Statistical analysis was performed using SPSS software version 25.0 (SPSS, Chicago, IL, USA). The Kolmogorov–Smirnov test was used to analyze the normal distribution of quantitative variables. The T-test was used for parametric data, and the Mann–Whitney-U test was used for nonparametric data. The Spearman correlation coefficient was used to investigate correlations. *p*-values < 0.05 were considered statistically significant. The Bonferroni method was used to adjust for multiple tests.

## 3. Results

Questionnaires of 80 patients were evaluated. Of these, 41 patients were male (51.2%), and 39 were female (48.8%). The mean age was 76.7 ± 9.4 years. The youngest patient was 51, and the oldest was 91 years. Forty patients (50.0%) were treated for systemic hypertension, and eight (10.0%) had diabetes.

Patients’ ophthalmological characteristics are presented in Table 1. A total of 49 patients (61.3%) had primary open angle glaucoma, 25 (31.3%) had pseudoexfoliation glaucoma, 4 patients had normal tension glaucoma (5.0%), and 2 patients had pigment dispersion glaucoma (2.5%). Nine patients had previous laser trabeculoplasty (11.3%). Nineteen patients (23.8%) had postoperative needling with 5-Fluoruracil, and nine patients (11.3%) had postoperative hyphema. Mean preoperative IOP was 23.7 ± 6.2 mmHg. One year after the operation, the IOP was reduced to 16.2 ± 4.8 mmHg, and the number of glaucoma medications needed was lowered to 1.53 ± 1.42.

Quality of life was assessed by GSS. The average score for visual symptoms was 71.3 ± 26.1 and 79.8 ± 17.7 for non-visual symptoms. The mean total score was 76.4 ± 17.9. The items with the lowest scores were “hard to see in daylight” (65.5 ± 36.3) and “hard to see in dark places” (66.5 ± 38.0). The items with the highest scores were “soreness/tiredness” (84.0 ± 27.4) and “burning/stinging” (82.0 ± 26.0). On average, the date of the survey was 1148 ± 386 days after the operation (range: 569–2295). There was no correlation of any item assessed with the number of days since the operation occurred or with age or mean defect (*p* > 0.05). The summarized score of visual symptoms correlated with postoperative visual acuity (r = 0.26; *p* = 0.033).

Patients were asked whether the operation led to a reduction in glaucoma medications. A total of 44 patients (55.0%) indicated that they needed the same amount of eye drops as before the operation, and 36 patients (45.0%) stated that they needed fewer eye drops. GSS scores separated by the reduction in glaucoma medication are depicted in Table 2. Overall GSS Scores were significantly different between groups (81.3 ± 14.6 vs. 72.4 ± 19.8; *p* = 0.028). Three vision-related items had significantly better scores in the group in which the operation led to a reduction in eye medication. “Hard to see in daylight” (75.0 ± 31.1 vs. 57.7 ± 39.1; *p* = 0.035), “hard to see in dark places” (81.1 ± 28.7 vs. 54.9 ± 41.2 *p* = 0.002), and “halos around lights” (88.3 ± 25.9 vs. 68.8 ± 38.6; *p* = 0.002) significantly differed between groups. There was no difference between groups regarding age (75.5 ± 9.8 years vs. 77.6 ± 9.1 years; *p* = 0.320), visual acuity (0.18 ± 0.37 LogMAR vs. 0.19 ± 0.21 LogMAR; *p* = 0.077), or mean defect (11.0 ± 7.3 dB vs. 10.3 ± 5.4 dB; *p* = 0.769).

Overall, satisfaction with the operation was high (7.6 ± 2.7). The scores for satisfaction correlated with the total GSS scores (r = 0.43; *p* < 0.001). Correlations between satisfaction and each item of the GSS score are indicated in Table 3. “Blurry/dim vision” (r = 0.53, *p* < 0.001) and “hard to see in daylight” (r = 0.34, *p* = 0.004) correlated significantly. Patients’ satisfaction with the operation did not correlate with age, mean defect, or the number of days since the operation (*p* > 0.05). Satisfaction was significantly higher when the operation led to a reduction in eyedrops (8.3 ± 2.0 vs. 6.8 ± 3.1; *p* = 0.034)

## 4. Discussion

We investigated the quality of life in glaucoma patients who underwent phacoemulsification combined with Xen^®^. After one year, the procedure demonstrated a sustained IOP reduction. We found that patients who needed fewer glaucoma medications also displayed a better quality of life. Patients with better quality of life scores were more satisfied with the operation.

All of our patients underwent phacoemulsification with Xen^®^. The IOP was reduced from 23.1 mmHg to 16.2 mmHg one year after the operation (*p* < 0.001). The IOP reduction of 6.9 mmHg is comparable with previous studies by Gilmann et al. [20,21]. In our study, the mean number of glaucoma medications was 1.53 one year after the operation, which is considerably higher than the 0.4 reported previously [20]. This difference may be due to different patient characteristics and selection criteria, as preoperative IOP were also different.

The mean total GSS score in our study cohort was 76.4 ± 17.9. The LIGHT Study reports a median GSS score of 85 for previously untreated primary open angle glaucoma (POAG) patients [22]. The lower GSS scores found for our patients are attributable to the fact that our patients were older (mean age 76.7 ± 9.4 years) than the patients of the LIGHT Study (mean age 64.0 ± 12.0 years), and all of our patients were already treated with topical glaucoma medications. Furthermore, patients in the LIGHT Study had milder visual field defects than our patients. Another study investigating the impact of a change to preservative-free eye drops on quality of life found a total GSS score of 65.4 at baseline, which improved to 76.6 four weeks after switching medication [23]. In glaucoma patients with visual field defects in only one eye, a GSS of 77.6 was found, which decreased to 73.1 in patients with visual field defects in both eyes [24]. Thus, the GSS scores found in our study are comparable with previously reported GSS scores.

Here, we report an increased quality of life in patients who needed fewer glaucoma medications than before the operation. This finding is in agreement with previous studies showing that the number of topical eye drops correlates well with ocular surface disease [25] and thus has an impact on quality of life [26]. In line with our findings, a previous study reported that GSS scores were significantly lower in patients requiring two or more eye drops per day [27]. In a study by Basilio et al. [28], GSS scores were compared between patients who underwent minimal invasive glaucoma surgery with Xen^®^ and trabeculectomy. They did not find a difference between the two groups, but they found a strong negative correlation between the administration of topical anti-hypertensive drugs and GSS scores. This is consistent with our results, as we found a significantly better quality of life for three vision-related items (“hard to see in daylight”, “hard to see in dark places”, and “halos around lights”) in patients requiring fewer glaucoma medications compared to before the operation. Basilio et al. only included patients who underwent Xen^®^ implantation as a standalone procedure, while in our study, all patients had a combined phacoemulsification with Xen^®^. When investigating each item separately, we found that the difference was statistically significant in vision-related items. This is not surprising as glaucoma medication leads to dry eye disease, which is characterized by a reduced break-up time, punctate superficial keratopathy, and finally, a reduced functional visual acuity [29]. In our study population, non-visual symptoms were not significantly better in the group with reduced glaucoma medication. This could have several reasons. First, as some non-visual items showed better scores in the reduced medication group, differences may have reached statistical significance with higher patient numbers. Second, we only asked for a reduction in glaucoma medications and not for the absolute number of eye drops. While a relative reduction of medication may be relevant for visual symptoms there may be a certain threshold for the number of eye drops for non-visual symptoms.

Patients’ satisfaction with the operation in our cohort was comparable with previous results after trabeculectomy and canaloplasty [30]. Dry eye disease has been identified as a major factor affecting postoperative patient satisfaction after phacoemulsification [31]. In this study, we found that two vision-related items (“blurry/dim vision” and “hard to see in daylight”) were significantly correlated with postoperative satisfaction. If the operation led to a reduction in glaucoma medications, satisfaction was significantly higher. This is further confirmed by previous studies showing that ocular surface disease is related to the number of glaucoma medications [32] and is significantly associated with glaucoma patients’ satisfaction [33].

Our study has several limitations. We did not evaluate whether patients were using preserved or preservative-free eye drops. Given the study’s design, we do not have data on visual acuity or dry-eye assessment at the time of the survey. Questionnaires rely on the subjective perception of patients. Furthermore, we investigated the quality of life at different stages postoperatively by a cross-sectional approach and we did not investigate the quality of life prior to surgery. We did not make ophthalmological examinations at the time of the survey and we will aim for a longitudinal approach in future studies.

In summary, we have found that the quality of life after phacoemulsification combined with Xen^®^ is higher when the operation leads to a reduction in glaucoma medications. Our findings demonstrate a beneficial effect of successful minimally invasive glaucoma surgery on patients’ quality of life.

## Figures and Tables

**Table 1:** Patient’s ophthalmological characteristics. OCT: Optical Coherence Tomography, IOP: Intraocular Pressure, LogMAR: Logarithm of the Minimum Angle of Resolution, SD: Standard Deviation.

	Mean	SD
Pachymetry (µm)	543.4	39.2
Mean defect (dB)	−10.5	−6.1
OCT global index (µm)	67.5	17.9
IOP preoperative (mmHg)	23.7	6.2
IOP 1 year after operation (mmHg)	16.2	4.8
Glaucoma medications preoperative (n)	2.53	1.13
Glaucoma medications 1 year after operation (n)	1.53	1.42
Visual acuity preoperative (LogMAR)	0.26	0.27
Visual acuity 1 year after operation (LogMAR)	0.19	0.29

**Table 2:** Glaucoma Symptom Score (GSS) results. Scores of all single items are depicted and separated by the reduction in eye medication. SD: Standard Deviation.

	Reduction Mean (SD) (*n* = 36)	No Reduction Mean (SD) (*n* = 43)	*p*-Value
Burning/stinging	87.2 (16.0)	77.2 (31.6)	0.316
Tearing	79.4 (23.2)	73.0 (32.6)	0.559
Dryness	74.4 (32.6)	76.7 (34.3)	0.527
Itching	85.0 (22.6)	77.7 (25.9)	0.163
Soreness/tiredness	82.8 (27.1)	84.2 (27.8)	0.761
Blurry/dim vision	81.1 (27.0)	70.2 (36.6)	0.239
Feeling of something in your eyes	78.3 (27.6)	83.3 (29.3)	0.217
Hard to see in daylight	75.0 (31.1)	57.7 (39.1)	**0.040**
Hard to see in dark places	81.1 (28.7)	54.9 (41.2)	**0.005**
Halos around light	88.3 (25.9)	68.8 (38.6)	**0.010**
Overall GSS Score	81.3 (14.6)	72.4 (19.8)	**0.028**

**Table 3:** Correlation of Glaucoma Symptom Scale (GSS) results with overall patient satisfaction. Spearman’s correlation coefficient and *p*-values are depicted.

	Spearman’s Correlation Coefficient	
	Rho	*p*-Value
Burning/stinging	0.12	0.344
Tearing	0.18	0.154
Dryness	0.12	0.321
Itching	0.11	0.395
Soreness/tiredness	0.18	0.146
Blurry/dim vision	**0.53**	**<0.001**
Feeling of something in your eyes	0.08	0.538
Hard to see in daylight	**0.34**	**0.004**
Hard to see in dark places	0.19	0.115
Halos around light	0.09	0.468

## Data Availability

The datasets generated during and/or analyzed during the current study are available from the corresponding author on reasonable request.

## References

[1] Wilson M.R., Coleman A.L., Yu F., Bing E.G., Sasaki I.F., Berlin K., Winters J., Lai A. (1998). Functional status and well-being in patients with glaucoma as measured by the Medical Outcomes Study Short Form-36 questionnaire. Ophthalmology.

[2] Janz N.K., Wren P.A., Lichter P.R., Musch D.C., Gillespie B.W., Guire K.E. (2001). Quality of life in newly diagnosed glaucoma patients: The Collaborative Initial Glaucoma Treatment Study. Ophthalmology.

[3] Kobelt G., Jonsson B., Bergstrom A., Chen E., Linden C., Alm A. (2006). Cost-effectiveness analysis in glaucoma: What drives utility? Results from a pilot study in Sweden. Acta Ophthalmol. Scand..

[4] Peters D., Heijl A., Brenner L., Bengtsson B. (2015). Visual impairment and vision-related quality of life in the Early Manifest Glaucoma Trial after 20 years of follow-up. Acta Ophthalmol..

[5] Aspinall P.A., Johnson Z.K., Azuara-Blanco A., Montarzino A., Brice R., Vickers A. (2008). Evaluation of quality of life and priorities of patients with glaucoma. Investig. Ophthalmol. Vis. Sci..

[6] Asiedu K., Abu S.L. (2019). The impact of topical intraocular pressure lowering medications on the ocular surface of glaucoma patients: A review. J. Curr. Ophthalmol..

[7] Ji H., Zhu Y., Zhang Y., Li Z., Ge J., Zhuo Y. (2016). Dry Eye Disease in Patients with Functioning Filtering Blebs after Trabeculectomy. PLoS ONE.

[8] Fechtner R.D., Godfrey D.G., Budenz D., Stewart J.A., Stewart W.C., Jasek M.C. (2010). Prevalence of ocular surface complaints in patients with glaucoma using topical intraocular pressure-lowering medications. Cornea.

[9] Di Staso S., Agnifili L., Cecannecchia S., Di Gregorio A., Ciancaglini M. (2018). In Vivo Analysis of Prostaglandins-induced Ocular Surface and Periocular Adnexa Modifications in Patients with Glaucoma. In Vivo.

[10] Benitez-Del-Castillo J., Labetoulle M., Baudouin C., Rolando M., Akova Y.A., Aragona P., Geerling G., Merayo-Lloves J., Messmer E.M., Boboridis K. (2017). Visual acuity and quality of life in dry eye disease: Proceedings of the OCEAN group meeting. Ocul. Surf..

[11] Koh S. (2016). Mechanisms of Visual Disturbance in Dry Eye. Cornea.

[12] Erb C., Gast U., Schremmer D. (2008). German register for glaucoma patients with dry eye. I. Basic outcome with respect to dry eye. Graefes Arch. Clin. Exp. Ophthalmol..

[13] Scelfo C., ElSheikh R.H., Shamim M.M., Abbasian J., Ghaffarieh A., Elhusseiny A.M. (2022). Ocular Surface Disease in Glaucoma Patients. Curr. Eye Res..

[14] Lewis R.A. (2014). Ab interno approach to the subconjunctival space using a collagen glaucoma stent. J. Cataract Refract. Surg..

[15] Gabbay I.E., Goldberg M., Allen F., Lin Z., Morley C., Pearsall T., Muraleedharan V., Ruben S. (2021). Efficacy and safety data for the Ab interno XEN45 gel stent implant at 3 Years: A retrospective analysis. Eur. J. Ophthalmol..

[16] Lee B.L., Gutierrez P., Gordon M., Wilson M.R., Cioffi G.A., Ritch R., Sherwood M., Mangione C.M. (1998). The Glaucoma Symptom Scale. A brief index of glaucoma-specific symptoms. Arch. Ophthalmol..

[17] Rossi G.C., Pasinetti G.M., Scudeller L., Milano G., Mazzone A., Raimondi M., Bordin M., Lanteri S., Bianchi P.E. (2013). The Italian version of the Glaucoma Symptom Scale Questionnaire: Translation, validation, and reliability. J. Glaucoma.

[18] Ruiz M.A., Pardo A., de la Casa J.M.M., Polo V., Esquirol J., Soto J. (2009). Cultural adaptation and validation into Spanish of the Glaucoma Symptom Scale (GSS). Med. Clin..

[19] Sencanic I., Gazibara T., Dotlic J., Stamenkovic M., Jaksic V., Bozic M., Grgurevic A. (2019). Glaucoma Symptom Scale: Psychometric properties of the Serbian version. PLoS ONE.

[20] Gillmann K., Bravetti G.E., Mermoud A., Rao H.L., Mansouri K. (2019). XEN Gel Stent in Pseudoexfoliative Glaucoma: 2-Year Results of a Prospective Evaluation. J. Glaucoma.

[21] Gillmann K., Bravetti G.E., Rao H.L., Mermoud A., Mansouri K. (2021). Combined and stand-alone XEN 45 gel stent implantation: 3-year outcomes and success predictors. Acta Ophthalmol..

[22] Konstantakopoulou E., Gazzard G., Vickerstaff V., Jiang Y., Nathwani N., Hunter R., Ambler G., Bunce C. (2018). The Laser in Glaucoma and Ocular Hypertension (LiGHT) trial. A multicentre randomised controlled trial: Baseline patient characteristics. Br. J. Ophthalmol..

[23] Abegao Pinto L., Vandewalle E., Gerlier L., Stalmans I., The CosoptUD Switch Study Group (2014). Improvement in glaucoma patient quality of life by therapy switch to preservative-free timolol/dorzolamide fixed combination. Ophthalmologica.

[24] Rulli E., Quaranta L., Riva I., Poli D., Hollander L., Galli F., Katsanos A., Oddone F., Torri V., Weinreb R.N. (2018). Visual field loss and vision-related quality of life in the Italian Primary Open Angle Glaucoma Study. Sci. Rep..

[25] Leung E.W., Medeiros F.A., Weinreb R.N. (2008). Prevalence of ocular surface disease in glaucoma patients. J. Glaucoma.

[26] Camp A., Wellik S.R., Tzu J.H., Feuer W., Arheart K.L., Sastry A., Galor A. (2015). Dry eye specific quality of life in veterans using glaucoma drops. Cont. Lens Anterior Eye.

[27] Rossi G.C.M., Tinelli C., Pasinetti G., Milano G., Bianchi P.E. (2009). Dry eye syndrome-related quality of life in glaucoma patients. Eur. J. Ophthalmol..

[28] Basilio A.L., Moura-Coelho N., Passos I., Cardoso M.S., Domingues I., Reina M., Flores R.M.R.P., Gomes T. (2018). XEN((R)) implant and trabeculectomy medium-term quality of life assessment and comparison of results. Int. J. Ophthalmol..

[29] Ishida R., Kojima T., Dogru M., Kaido M., Matsumoto Y., Tanaka M., Goto E., Tsubota K. (2005). The application of a new continuous functional visual acuity measurement system in dry eye syndromes. Am. J. Ophthalmol..

[30] Klink T., Sauer J., Korber N.J., Grehn F., Much M.M., Thederan L., Matlach J., Salgado J.P. (2015). Quality of life following glaucoma surgery: Canaloplasty versus trabeculectomy. Clin. Ophthalmol..

[31] Schallhorn S.C., Hettinger K.A., Teenan D., Venter J.A., Hannan S.J., Schallhorn J.M. (2020). Predictors of Patient Satisfaction After Refractive Lens Exchange With an Extended Depth of Focus IOL. J. Refract. Surg..

[32] Skalicky S.E., Goldberg I., McCluskey P. (2012). Ocular surface disease and quality of life in patients with glaucoma. Am. J. Ophthalmol..

[33] Lemij H.G., Hoevenaars J.G., van der Windt C., Baudouin C. (2015). Patient satisfaction with glaucoma therapy: Reality or myth?. Clin. Ophthalmol..

